# Functional parameters of small airways can guide bronchodilator use in idiopathic pulmonary fibrosis

**DOI:** 10.1038/s41598-020-75597-2

**Published:** 2020-10-29

**Authors:** Po-Wei Hu, Hsin-Kuo Ko, Kang-Cheng Su, Jia-Yih Feng, Wei-Juin Su, Yi-Han Hsiao, Diahn-Warng Perng

**Affiliations:** 1 Department of Chest Medicine, Taipei Veterans General Hospital, No. 201, Shipai Rd., Beitou District, Taipei, 11217 Taiwan, ROC; 2 Faculty of Medicine, School of Medicine, National Yang-Ming University, Taipei, Taiwan, ROC; 3 Department of Physiology, School of Medicine, National Yang-Ming University, Taipei, Taiwan, ROC

**Keywords:** Diseases, Respiratory tract diseases

## Abstract

Idiopathic pulmonary fibrosis (IPF) may present comorbid obstructive lung diseases with small airway dysfunction (SAD). Existing guidelines suggest that inhaled bronchodilators should be used if the ratio of forced expiratory volume in the 1st second and forced vital capacity (FEV_1_/FVC) < 0.7 in IPF. However, most IPF patients have FEV_1_/FVC > 0.7 even with coexisting emphysema. We retrospectively enrolled IPF patients who were registered at our outpatient clinic. At baseline, 63 patients completed computed tomography (CT) scans, lung function measurements, and symptom questionnaires. Among these patients, 54 (85.71%) underwent antifibrotic treatment and 38 (60.32%) underwent long-acting bronchodilator treatment. The median FEV_1_/FVC was 0.86. Not all patients treated with bronchodilators showed significant changes in lung function. IPF patients with SAD, determined by IOS parameters, showed significant improvement in FEV_1_, FEF_25–75%_, and symptom scores after bronchodilator treatment. Bronchodilator efficacy was not observed in patients without SAD. CT-confirmed emphysema was seen in 34.92% of patients. There were no changes in lung function or symptom scores after bronchodilator treatment in patients with emphysema. In conclusion, FEV_1_/FVC cannot reflect the airflow limitation in IPF. Emphysema in IPF is not a deciding factor in whether patients should receive bronchodilator treatment. IOS parameters may be useful to guide bronchodilator therapy in patients with IPF coexisting with SAD.

## Introduction

Idiopathic pulmonary fibrosis (IPF) is the most common idiopathic interstitial pneumonia; its chief characteristics are progressive aberrant deposition of the extracellular matrix leading to extensive lung remodeling^1,2^. The diagnosis of IPF is mainly based on the typical features of the usual interstitial pneumonia (UIP) pattern seen on high-resolution computed tomography (HRCT), although some patients with suspected IPF may need to have the histopathological UIP pattern confirmed by surgical lung biopsy or other invasive procedures^3^. The median survival with IPF is approximately 3 years from the time of diagnosis^4^. However, a recent report stated that IPF-related mortality is increasing across the European Union^5^.

The alterations of lung mechanics in IPF include reductions in lung compliance and volumes, impaired pulmonary gas exchange, reduced diffusing capacity, and increased pulmonary hemodynamics^6^. These changes may contribute to dyspnea, exercise limitation, and hypoxemia. The comorbidities can worsen the IPF patient’s lung function and survival outcomes, especially when combined with chronic obstructive lung diseases and emphysema^7^. The prevalence of chronic obstructive pulmonary disease (COPD), including emphysema, ranges from 6 to 67% and varies widely among countries and regions^7^. Emphysema is easily identified by HRCT. The presence of a post-bronchodilator ratio of forced expiratory volume in the 1st second and forced vital capacity (FEV_1_/FVC) < 0.7 is required to make a diagnosis of COPD. The reduced lung volume and resistance of the conducting airways in IPF lead to a higher-than-normal FEV_1_/FVC^8^. This makes diagnosing COPD in patients with IPF extremely difficult.

Dyspnea and exercise limitation are the major symptoms of both COPD and IPF. Bronchodilator therapy is recommended in COPD because it can ameliorate breathlessness and improve FEV_1_ and FVC^9^. In IPF combined with emphysema, it is suggested that inhaled bronchodilators should be used if airflow obstruction is present^10^. In one IPF cohort, the post-bronchodilator FEV_1_/FVC was 0.83 as FVC was reduced in proportion to total lung capacity. Among the patients in that study, 14.2% and 8.7% were diagnosed with COPD and asthma, respectively; 30% received bronchodilator medications^11^. Currently, to the best of our knowledge, there is no specific measurement to guide bronchodilator therapy in patients with IPF coexisting with obstructive lung diseases.

Impulse oscillometry (IOS) enables clinicians to assess respiratory mechanics during spontaneous breathing^12^. In contrast to spirometry, IOS is an effort-independent method that is convenient and more sensitive to detect small airway dysfunction (SAD); moreover, it correlates with the symptoms and disease severity of asthma and COPD^13,14^. This study aimed to investigate the functional parameters of small airways measured using IOS to determine whether these parameters can guide bronchodilator therapy in IPF patients.

## Methods

### Study design and data collection

This retrospective cohort study reviewed the medical records of adult patients (≥ 40 years of age) diagnosed with IPF based on the criteria provided by the American Thoracic Society (ATS)/European Respiratory Society (ERS)/Japanese Respiratory Society (JRS)/Latin American Thoracic Society (ALAT)^3,4^ in the Taipei Veterans General Hospital (TVGH) and registered in the Taiwan IPF cohort from October 1, 2017 to October 31, 2019. Data on baseline demographic variables were collected, including sex, age, smoking status, symptom scores (St. George Respiratory Questionnaire, SGRQ and COPD assessment score, CAT score)^15,16^, the presence of emphysema on HRCT, lung function parameters [including spirometry, IOS, diffusing capacity for carbon monoxide (*D*_LCO_) and six-minute walk test (6MWT)]. The SGRQ and CAT scores were measured as done in previous studies to evaluate the quality of life and symptoms in patients with IPF^17–21^. The patients were followed up regularly for lung function and symptom evaluation. Medications including bronchodilators and antifibrotic agents were prescribed based on clinicians’ judgment and reimbursement by the national health insurance in Taiwan. Bronchodilators included long-acting muscarinic antagonist (LAMA), long-acting beta-2 agonist (LABA), and inhaled corticosteroid (ICS); LAMA/LABA or LAMA/LABA/ICS combinations; and the antifibrotic agents included nintedanib and pirfenidone. The medical records and HRCT were reviewed by two independent pulmonology specialists with assistance from a third specialist in case of disagreement. Our study was carried out in accordance with the principles of the Declaration of Helsinki, and the protocol was approved by the Institutional Review Board of TVGH (VGHIRB No. 2017-06-007AC). Informed consent was obtained from all participants and/or their legal guardians.

### Pulmonary function tests

Pulmonary function tests including spirometry, *D*_LCO_, and 6MWT were performed on all patients. A standardized examination protocol was followed according to the ATS/ERS recommendations^22–24^, and details are described in the online Supplementary information. The interpretation of lung function tests was based on the recommendations of the ATS/ERS guidelines^25^.

### Impulse oscillometry

IOS was conducted using combined spirometry and IOS equipment (Jaeger MS-IOS Germany). A standardized examination was conducted on all patients according to the protocols of the ERS^26^ (detailed description in the online Supplementary information). We evaluated the following IOS parameters: difference in resistance at 5 Hz and 20 Hz (R_5_–R_20_), reactance at 5 Hz (X_5_), resonant frequency (Fres), and area under reactance curve between 5 Hz and resonant frequency (AX).

### Statistical analysis

The distribution of variables was assessed using the Kolmogorov–Smirnov goodness-of-fit test. Variables are expressed as mean ± standard deviation or median (interquartile range, IQR), unless otherwise specified. The Mann–Whitney U test and Pearson’s Chi-square-test were used for comparisons, as appropriate. To examine the relationships between measures, Pearson’s correlation coefficient (r) was used, when appropriate. A value of *p* < 0.05 was considered significant.

## Results

### Characteristics of study subjects

A total of 63 patients who had completed CT scans, lung function measurement, IOS and symptom questionnaires at baseline were enrolled in this study (Table 1). The median follow-up was 14 weeks. Among the patients, 85.71% (n = 54) received anti-fibrotic treatment, including nintedanib (n = 45) and pirfenidone (n = 9). In addition, 60.31% (n = 38) received bronchodilator treatment, including LAMA (n = 4), LAMA/LABA (n = 12), ICS/LABA (n = 8) and LAMA/LABA/ICS (n = 14). Only 4.76% (n = 3) showed airflow obstruction in the form of FEV_1_/FVC < 0.7. The median FEV_1_/FVC ratio was 0.86. The medial values of all IOS parameters (R_5_-R_20_, X_5_, Fres and AX) were worse than those we previously reported in healthy subjects^27^. Bronchodilator treatment was based on the physician’s judgment. IPF patients treated with bronchodilators had significantly lower FEV_1_% and FVC% as well as worse symptoms (SGRQ and CAT score) than those without bronchodilator treatment (Table 1). There were no differences in FEV_1_/FVC, FEF_25%-75%_, oxygen saturation (SaO_2_) at baseline or during the 6MWT, *D*_LCO_, or IOS parameters between patients with and without bronchodilator treatment. In addition, patients treated with bronchodilators did not have significant differences in lung function except the CAT score (Table 2).

**Table 1:** Baseline characteristics of patients.

Characteristics			Total (N = 63)	BD Rx (−) (N = 25)	BD Rx (+) (N = 38)	*p* value
Age (years)			77 (69 to 86)	80 (70 to 86)	75 (68 to 85)	0.35
Smoker (%)			33 (52.38%)	13 (52.00%)	20 (52.63%)	0.96
Male sex (%)			54 (85.71%)	21 (84.00%)	33 (86.84%)	0.75
**SGRQ**			22.12 (16.38 to 33.36)	18.59 (14.00 to 23.40)	25.09 (17.87 to 42.59)	0.02
Symptom domain			30.32 (16.76 to 39.79)	33.71 (15.42 to 41.79)	30.17 (21.13 to 39.43)	0.90
Activity domain			47.23 (23.30 to 59.46)	29.31 (17.14 to 47.24)	53.23 (29.00 to 71.37)	0.01
Impact domain			8.80 (4.06 to 22.93)	7.15 (2.96 to 14.78)	10.58 (4.20 to 29.65)	0.08
CAT score			7 (4 to 11)	5.00 (3.00 to 7.50)	9.00 (4.25 to 12.00)	0.03
Baseline SaO_2_ in 6MWT			95.00 (93.00 to 96.00)	95.00 (93.00 to 96.00)	95.00 (94.00 to 96.00)	0.82
SaO_2_ drop during 6MWT			5.00 (3.00 to 8.50)	5.00 (1.00 to 7.00)	6.00 (4.00 to 9.00)	0.13
Patients with a FEV_1_/FVC < 0.7 (%)			3 (4.76%)	0 (0.00%)	3 (7.89%)	0.15
HRCT-defined emphysema (%)			22 (34.92%)	7 (28.00%)	15 (39.47%)	0.35
**Antifibrotics treatment (%)**			54 (85.71%)	20 (80.00%)	34 (89.47%)	0.51
No			9 (14.29%)	5 (20.00%)	4 (10.53%)	0.29
Nintedanib			45 (71.43%)	16 (64.00%)	29 (76.32%)	0.29
Pirfenidone			9 (14.29%)	4 (16.00%)	5 (13.16%)	0.72
FVC (L)			2.01 (1.74 to 2.39)	2.26 (1.85 to 2.68)	1.97 (1.72 to 2.31)	0.18
FVC (% predicted value)			70.00 (57.00 to 81.00)	76.00 (64.00 to 86.00)	64.00 (56.00 to 74.00)	0.02
FEV_1_ (L)			1.73 (1.47 to 1.98)	1.93 (1.58 to 2.36)	1.72 (1.43 to 1.91)	0.20
FEV_1_ (% predicted value)			83.00 (70.00 to 99.00)	97.00 (81.00 to 110.00)	78.00 (68.00 to 88.00)	0.02
FEV_1_/FVC			0.86 (0.82 to 0.91)	0.86 (0.82 to 0.89)	0.86 (0.82 to 0.93)	0.54
FEF_25–75%_ (L/s)			2.38 (1.55 to 3.41)	2.63 (1.85 to 3.17)	2.22 (1.49 to 3.44)	0.77
FEF_25–75%_ (% predicted value)			92.00 (67.00 to 114.00)	93.00 (78.00 to 119.00)	89.00 (64.00 to 113.00)	0.74
*D*_LCO_ (% predicted value)			34.00 (23.00 to 47.00)	39.00 (26.00 to 50.00)	33.00 (23.00 to 40.00)	0.19
R_5_-R_20_ (kPa L(− 1)sec)			0.08 (0.06 to 0.12)	0.08 (0.07 to 0.12)	0.09 (0.06 to 0.12)	0.79
X_5_ (kPa L(− 1)sec)			− 0.15 (− 0.20 to − 0.12)	− 0.15 (− 0.19 to − 0.12)	− 0.15 (− 0.20 to − 0.12)	0.98
AX (kPa L(− 1))			0.69 (0.48 to 1.07)	0.72 (0.41 to 1.09)	0.69 (0.52 to 1.03)	0.68
Fres (Hz)			16.10 (14.72 to 17.80)	15.87 (13.99 to 17.94)	16.21 (15.07 to 17.66)	0.34

The data are described as number (%) for categorical variables, and median (interquartile range, IQR) for non-normally distributed continuous variables. *p* values were generated from the Mann–Whitney U test for two-group (with versus without bronchodilator treatment) comparisons.

*BD Rx* bronchodilator treatment, *SGRQ* St. George Respiratory Questionnaire, *CAT* COPD assessment test, *SaO*_*2*_ oxygen saturation (%), *6MWT* six-minute walk test, *FEV*_*1*_ forced expiratory volume in the 1st second, *FVC* forced vital capacity, *HRCT* high-resolution computed tomography, *FEF*_*25–75%*_ forced expiratory flow after expiration of 25–75% of forced vital capacity, *DLCO* diffusing capacity for carbon monoxide, *R5* resistance at 5 Hz, *R20* resistance at 20 Hz, *X5* reactance at 5 Hz, *Fres* resonant frequency, *AX* area of reactance.

**Table 2:** Differences in parameters between patients with versus without bronchodilator treatment.

		BD Rx (−) (N = 25)	BD Rx ( +) (N = 38)	*p* value
Δ FVC (L)		**− **0.09 (− 0.17 to 0.09)	0.03 (− 0.11 to 0.14)	0.13
Δ FEV_1_ (L)		− 0.03 (− 0.19 to 0.07)	0.03 (− 0.04 to 0.1)	0.08
Δ FEF_25–75%_ (L/s)		− 0.07 (− 0.5 to 0.23)	0.06 (− 0.35 to 0.47)	0.36
Δ DLCO (% predicted value)		− 5.00 (− 11.00 to 0.00)	0.00 (− 8.50 to 3.00)	0.12
Δ R_5_-R_20_ (kPa L(− 1)sec)		0.01 (− 0.01 to 0.04)	0.00 (− 0.03 to 0.03)	0.17
Δ X_5_ (kPa L(− 1)sec)		− 0.01 (− 0.03 to 0.02)	− 0.01 (− 0.04 to 0.03)	0.94
Δ AX (kPa L(− 1))		0.07 (− 0.08 to 0.3)	0.05 (− 0.07 to 0.19)	0.75
Δ Fres (Hz)		0.05 (− 0.72 to 1.89)	0.08 (− 1.13 to 1.95)	0.72
Δ CAT score		1.00 (− 1.50 to 4.50)	− 2.00 (− 6.00 to 0.00)	0.01
**Δ SGRQ**		2.28 (− 4.64 to 11.5)	− 1.28 (− 11.43 to 6.37)	0.18
Δ Symptom domain		− 2.95 (− 13.27 to 1.31)	− 5.33 (− 15.05 to 12.9)	0.53
Δ Activity domain		6.21 (− 8.93 to 27.39)	0.00 (− 12.36 to 11.62)	0.08
Δ Impact domain		0.21 (− 4.47 to 7.75)	− 0.69 (− 13.64 to 7.15)	0.34

The data are described as median (interquartile range, IQR) for non-normally distributed continuous variables. *p* values were generated from the Mann–Whitney U test for two-group comparisons.

*BD Rx* bronchodilator treatment, *△* difference between visit 1 and visit 2, *FVC* forced vital capacity, *FEV*_*1*_ forced expiratory volume in the 1st second, *FEF*_*25–75%*_ forced expiratory flow after expiration of 25% to 75% of forced vital capacity, *DLCO* diffusing capacity for carbon monoxide, *R5* resistance at 5 Hz, *R20* resistance at 20 Hz, *X5* reactance at 5 Hz, *Fres* resonant frequency, *AX* area of reactance, *CAT* COPD assessment test, *SGRQ* St. George Respiratory Questionnaire.

### Baseline characteristics of IPF patients with or without emphysema

Among the patients, 34.92% (n = 22) had CT scan-confirmed emphysema (Table 3). All patients with emphysema were male. The incidence of smoking history and male sex among IPF patients with emphysema was significantly higher than among those without emphysema. In the emphysema group, 27.27% (n = 6) of patients were never smokers and had no history of occupational or environmental exposure. The FEV_1_/FVC and FEF_25–75%_ were significantly lower in the IPF with emphysema group. The FEV_1_, FVC, *D*_LCO_, IOS parameters and symptom scores were not different between two groups. Although emphysema might indicate the coexistence of air trapping, it was hard to diagnose them with a coexisting COPD since their FEV_1_/FVC was not less than 0.7 (Table 3), which is required to diagnose COPD according to the GOLD guideline^9^.

**Table 3:** Baseline characteristics of patients with versus without emphysema.

Characteristics (total N = 63)			Emphysema ( +) (N = 22)	Emphysema (−) (N = 41)	*p* value
Age (years)			76.00 (70.25 to 86.00)	77.00 (69.00 to 86.00)	0.95
Smoker (%)			16 (72.73%)	17 (41.46%)	0.02
Male sex (%)			22 (100.00%)	32 (78.05%)	0.02
**SGRQ**			21.69 (12.85 to 36.62)	22.93 (16.61 to 29.87)	0.83
Symptom domain			30.17 (13.72 to 38.3)	30.49 (21.57 to 44.02)	0.42
Activity domain			53.16 (17.14 to 67.02)	41.39 (27.52 to 55.08)	0.99
Impact domain			6.36 (3.99 to 25.12)	8.97 (4.06 to 17.73)	0.79
CAT score			6.00 (4.00 to 12.00)	7.50 (4.00 to 11.00)	0.48
Baseline SaO_2_ in 6MWT			95.00 (92.50 to 95.00)	96.00 (93.00 to 97.00)	0.07
SaO_2_ drop during 6MWT			5.00 (4.00 to 6.75)	5.00 (3.00 to 9.00)	0.62
Bronchodilator treatment			15 (68.18%)	23 (56.1%)	0.35
**Antifibrotics treatment (%)**			19 (86.36%)	35 (85.37%)	0.99
No			3 (13.64%)	6 (14.63%)	0.91
Nintendanib			16 (72.73%)	29 (70.73%)	0.87
Pirfenidone			3 (13.64%)	6 (14.63%)	0.97
FVC (L)			2.13 (1.92 to 2.39)	1.95 (1.69 to 2.33)	0.13
FVC (% predicted value)			73.50 (60.50 to 82.25)	69.00 (56.00 to 77.00)	0.33
FEV_1_ (L)			1.75 (1.60 to 1.93)	1.68 (1.42 to 2.03)	0.93
FEV_1_ (% predicted value)			82.00 (69.25 to 99.00)	83.00 (71.00 to 99.00)	0.76
FEV_1_/FVC			81.00 (71.50 to 83.75)	88.00 (85.00 to 92.00)	< 0.01
FEF_25–75%_ (L/s)			1.71 (1.08 to 2.42)	2.65 (1.91 to 3.63)	< 0.01
FEF_25–75%_ (% predicted value)			75.00 (40.00 to 92.00)	106.50 (80.00 to 124.00)	< 0.01
DLCO (% predicted value)			30.50 (22.25 to 41.00)	37.00 (26.00 to 47.00)	0.18
R_5_–R_20_ (kPa L(− 1)sec)			0.09 (0.06 to 0.12)	0.08 (0.07 to 0.12)	0.77
X_5_ (kPa L(− 1)sec)			− 0.15 (− 0.20 to − 0.11)	− 0.15 (− 0.17 to − 0.12)	0.94
AX (kPa L(− 1))			0.74 (0.42 to 1.25)	0.68 (0.50 to 1.04)	0.89
Fres (Hz)			15.60 (14.70 to 17.53)	16.29 (15.00 to 17.94)	0.82

The data are described as number (%) for categorical variables and the median (interquartile range, IQR) for non-normally distributed continuous variables. *p* values were generated from the Mann–Whitney U test for two-group comparisons.

*SGRQ* St. George Respiratory Questionnaire, *CAT* COPD assessment test, *SaO*_*2*_ oxygen saturation (%), *6MWT* six-minute walk test, *FEV1* forced expiratory volume in the 1st second, *FVC* forced vital capacity, *FEF*_*25–75%*_ forced expiratory flow after expiration of 25% to 75% of forced vital capacity, *DLCO* diffusing capacity for carbon monoxide, *R5* resistance at 5 Hz, *R20* resistance at 20 Hz, *X5* reactance at 5 Hz, *Fres* resonant frequency, *AX* area of reactance.

### Correlation between exercise desaturation, symptoms, lung function and small airway parameters

Among all patients, *D*_LCO_ was significantly associated with SGRQ score and its activity domain score. FVC%, FEV_1_%, and FEF_25–75%_ were not correlated with SGRQ score or CAT score (Supplementary Table S1). The correlations between IOS parameters, lung function and symptom scores are shown in Supplementary Table S2. Oxygen desaturation during the 6MWT at baseline was significantly associated with FVC%, FEV_1_%, *D*_LCO_, SGRQ score, and the SGRQ activity domain score. The IOS parameter AX was correlated with percentage of predicted FEV_1_ and FEF_25–75%_ values and SGRQ activity domain score (Supplementary Table S2). Other IOS parameters, including R_5_–R_20_, X_5_ and Fres did not simultaneously correlate with lung function parameters and symptom scores.

### Bronchodilator efficacy in IPF according to coexisting emphysema

The bronchodilator efficacy in IPF patients with (n = 22) or without (n = 41) emphysema is shown in Table 4. In IPF patients with emphysema, there were no significant differences in terms of spirometry, *D*_LCO_, IOS parameters or symptom score between patients with (n = 15) and without (n = 7) bronchodilator treatment in the 14-week follow-up period. In patients without emphysema who received bronchodilator treatment (n = 23), there were significant improvements in the CAT score and SGRQ activity domain score compared to those in patients without bronchodilator treatment (n = 18), while no differences were observed in the changes in pulmonary function or IOS parameters. We therefore conclude that emphysema cannot be a deciding factor in whether patients should receive bronchodilator treatment.

**Table 4:** The effect of bronchodilator treatment in patients with versus without emphysema.

		Emphysema ( +) (N = 22)			Emphysema (−) (N = 41)		
		BD Rx (−) (N = 7)	BD Rx ( +) (N = 15)	*p* value	BD Rx (−) (N = 18)	BD Rx ( +) (N = 23)	*p* value
FVC (% predicted value)		80.00 (70.00 to 81.50)	66.00 (60.00 to 84.00)	0.62	75.00 (65.75 to 89.50)	62.00 (54.50 to 71.50)	0.01
FEV_1_ (% predicted value)		96.00 (76.00 to 100.50)	77.00 (69.50 to 92.50)	0.55	97.5 (81.25 to 110.75)	79.00 (67.00 to 87.00)	0.02
FEF_25–75%_(% predicted value)		62.00 (40.00 to 80.03)	76.00 (45.50 to 100.50)	0.42	108.00 (85.00 to 148.00)	105.00 (79.00 to 117.00)	0.54
FEV_1_/FVC %		80.00 (79.50 to 82.00)	83.00 (70.00 to 88.00)	0.72	88.50 (85.25 to 90.00)	88.00 (84.50 to 93.50)	0.56
*D*_LCO_ (% predicted value)		31.00 (26.00 to 41.00)	30.00 (19.50 to 40.00)	0.92	44.50 (26.50 to 53.75)	34.00 (25.50 to 40.00)	0.21
R_5_-R_20_ (kPa L(-1)sec)		0.09 (0.07 to 0.12)	0.08 (0.06 to 0.12)	1.00	0.08 (0.07 to 0.11)	0.09 (0.06 to 0.12)	0.69
X_5_ (kPa L(-1)sec)		− 0.17 (− 0.22 to − 0.13)	− 0.15 (− 0.19 to − 0.11)	0.44	− 0.15 (− 0.17 to − 0.12)	− 0.15 (− 0.19 to − 0.13)	0.55
AX (kPa L(-1))		1.09 (0.59 to 1.47)	0.68 (0.43 to 0.88)	0.27	0.64 (0.42 to 1.03)	0.69 (0.59 to 1.10)	0.21
Fres (Hz)		16.97 (15.36 to 19.45)	15.31 (14.62 to 17.11)	0.27	15.44 (13.87 to 17.25)	16.43 (15.83 to 19.21)	0.06
CAT score		5.00 (3.00 to 7.00)	10.00 (4.00 to 14.25)	0.34	5.00 (3.00 to 7.25)	8.50 (6.50 to 12.00)	0.05
**SGRQ**		18.59 (9.92 to 23.53)	26.93 (15.68 to 51.32)	0.20	18.45 (15.84 to 23.38)	25.09 (19.48 to 39.23)	0.02
Symptom domain		13.72 (10.89 to 36.50)	30.53 (27.61 to 39)	0.18	34.45 (29.43 to 44.97)	28.88 (19.12 to 39.67)	0.27
Activity domain		17.14 (14.15 to 53.23)	53.39 (18.69 to 76.02)	0.19	29.41 (17.14 to 42.85)	50.57 (29.56 to 68.26)	0.01
Impact domain		4.16 (3.65 to 14.78)	10.83 (4.06 to 42.13)	0.31	7.43 (1.83 to 13.76)	10.58 (6.46 to 27.26)	0.11
△FVC (L)		− 0.04 (− 0.26 to 0.14)	0.08 (− 0.06 to 0.18)	0.46	− 0.09 (− 0.16 to 0.03)	0.01 (− 0.13 to 0.11)	0.24
△FEV_1_ (L)		− 0.02 (− 0.12 to 0.09)	0.04 (− 0.01 to 0.10)	0.40	− 0.05 (− 0.19 to 0.06)	0.01 (− 0.06 to 0.09)	0.13
△FEF_25–75%_ (L/s)		0.03 (− 0.21 to 0.41)	0.05 (− 0.23 to 0.46)	0.95	− 0.23 (− 0.58 to 0.16)	0.06 (− 0.38 to 0.52)	0.27
△*D*_LCO_ (% predicted value)		0.00 (− 3.00 to 2.50)	1.00 (0.00 to 8.50)	0.36	0.02 (− 0.01 to 0.04)	− 5.00 (− 9.50 to 1.00)	0.32
△R_5_–R_20_ (kPa L(− 1)sec)		0.01 (− 0.01 to 0.06)	0.01 (− 0.03 to 0.03)	0.40	0.00 (− 0.02 to 0.02)	0.00 (− 0.03 to 0.02)	0.34
△X_5_ (kPa L(− 1)sec)		− 0.03 (− 0.06 to − 0.03)	0.01 (− 0.04 to 0.04)	0.20	0.02 (− 0.08 to 0.20)	− 0.01 (− 0.04 to 0.02)	0.35
△AX (kPa L(− 1))		0.26 (0.02 to 0.49)	0.00 (− 0.16 to 0.16)	0.17	0.46 (− 0.53 to 1.77)	0.11 (− 0.04 to 0.24)	0.54
△Fres (Hz)		− 0.55 (− 1.90 to 0.74)	0.41 (− 0.93 to 2.14)	0.41	1.00 (− 0.50 to 5.25)	− 0.1 (− 1.33 to 1.65)	0.28
△CAT score		2.00 (− 1.50 to 3.00)	− 1.00 (− 5.00 to 3.00)	0.80	2.73 (− 6.01 to 12.31)	− 3.00 (− 6.25 to − 1.00)	0.02
**△SGRQ**		1.41 (− 2.51 to 6.49)	3.60 (− 5.30 to 8.82)	1.00	0.02 (− 0.01 to 0.04)	− 3.97 (− 11.50 to 5.37)	0.16
△Symptom domain		− 0.42 (− 5.16 to 1.31)	− 3.39 (− 6.81 to 12.9)	0.88	− 3.65 (− 15.87 to 1.06)	− 7.29 (− 16.57 to 11.65)	0.58
△Activity domain		0.00 (− 8.93 to 13.28)	0.00 (− 12.36 to 11.83)	0.84	14.44 (− 5.04 to 32.38)	0.16 (− 13.01 to 8.04)	0.04
△Impact domain		− 0.05 (− 5.54 to 9.79)	2.52 (− 3.99 to 13.39)	0.88	0.36 (− 3.92 to 4.64)	− 1.68 (− 14.20 to 2.63)	0.14

The data are described as median (interquartile range, IQR) for non-normally distributed variables. *p* values were generated from the Mann–Whitney U test for two-group comparison.

*BD Rx* bronchodilator treatment, *FVC* forced vital capacity, *FEV1* forced expiratory volume in the 1st second, *FEF*_*25–75%*_ forced expiratory flow after expiration of 25% to 75% of forced vital capacity, *DLCO* diffusing capacity for carbon monoxide, *R5* resistance at 5 Hz, *R20* resistance at 20 Hz, *X5* reactance at 5 Hz, *Fres* resonant frequency, *AX* area of reactance, *CAT* COPD assessment test, *SGRQ* St. George Respiratory Questionnaire, *△* difference between visit 1 and visit 2.

### Bronchodilator efficacy in IPF based on small airway dysfunction

The bronchodilator efficacy in IPF patients with versus without SAD is shown in Table 5. We defined SAD according to the IOS parameter AX > 0.44 (kPa/L) at baseline^27^. In IPF patients with SAD (79.36%, n = 50), there was significant improvement in FEV_1_, FEF_25%-75%_, and CAT score after bronchodilator treatment. A trend of an increase in FVC (*p* = 0.06) was observed. The bronchodilator efficacy in patients with SAD defined by R_5_–R_20_ > 0.07 (kPa L(−1)sec), X_5_ < − 0.12 (kPa L(−1)sec) or Fres > 14.14Hz^27^ is shown in Supplementary Tables S3–S5. Patients without SAD did not achieve statistical improvement within the follow-up interval. Table 6 summarizes bronchodilator efficacy in IPF patients based on SAD defined according to different cutoffs of IOS parameters. In patients with R_5_–R_20_-defined SAD, there was also a significant improvement in FEV_1_, FEF_25–75%_, and CAT score after bronchodilator treatment.

**Table 5:** The effect of bronchodilator treatment in patients with versus without SAD.

			SAD ( +) (N = 50)			SAD (−) (N = 13)		
			BD Rx (−) (N = 18)	BD Rx ( +) (N = 32)	*p* value	BD Rx (−) (N = 7)	BD Rx ( +) (N = 6)	*p* value
FVC (% predicted value)			75.00 (64.00 to 82.50)	62.00 (55.75 to 73.00)	0.03	80.00 (77.00 to 93.50)	89.00 (68.75 to 92.00)	1.00
FEV_1_ (% predicted value)			96.50 (75.75 to 107.25)	76.50 (66.75 to 83.75)	0.04	102.00 (88.00 to 106.50)	101.00 (86.00 to 120.50)	0.94
FEF_25–75%_ (% predicted value)			89.50 (68.50 to 130.00)	89.00 (66.00 to 111.75)	0.63	93.00 (84.00 to 102.50)	94.00 (63.50 to 205.50)	0.83
FEV_1_/FVC %			86.50 (80.50 to 90.00)	86.00 (82.75 to 92.50)	0.61	85.00 (82.00 to 87.50)	85.50 (71.75 to 91.75)	0.89
*D*_LCO_ (% predicted value)			35.50 (26.50 to 47.50)	32.50 (26.00 to 40.00)	0.44	50.00 (31.50 to 59.50)	29.00 (17.00 to 47.75)	0.43
R_5_–R_20_ (kPa L(− 1)sec)			0.09 (0.08 to 0.12)	0.09 (0.07 to 0.14)	1.00	0.06 (0.06 to 0.07)	0.06 (0.04 to 0.07)	0.66
X_5_ (kPa L(− 1)sec)			− 0.17 (− 0.21 to − 0.15)	− 0.16 (− 0.22 to − 0.14)	0.56	− 0.11 (− 0.12 to − 0.08)	− 0.11 (− 0.12 to − 0.10)	1.00
AX (kPa L(− 1))			1.02 (0.69 to 1.35)	0.73 (0.61 to 1.20)	0.56	0.41 (0.38 to 0.41)	0.40 (0.34 to 0.42)	0.94
Fres (Hz)			16.83 (15.68 to 18.18)	16.47 (15.81 to 19.09)	0.98	13.99 (13.82 to 14.18)	14.46 (14.18 to 14.69)	0.23
CAT score			5.00 (3.00 to 7.25)	9.00 (4.00 to 12.00)	0.04	5.00 (2.50 to 6.50)	9.00 (5.00 to 9.00)	0.61
**SGRQ**			18.78 (15.97 to 23.89)	24.95 (17.47 to 50.80)	0.11	16.44 (11.36 to 20.00)	31.73 (22.12 to 36.62)	0.07
Symptom domain			30.25 (13.98 to 36.34)	30.17 (20.91 to 39.23)	0.88	38.13 (27.28 to 44.25)	38.30 (27.71 to 40.17)	1.00
Activity domain			35.25 (17.14 to 53.20)	47.84 (28.91 to 73.04)	0.10	17.14 (8.20 to 32.31)	53.62 (53.16 to 59.46)	0.02
Impact domain			8.24 (2.20 to 14.93)	10.86 (4.60 to 39.24)	0.13	4.16 (3.86 to 8.76)	6.36 (4.06 to 25.12)	0.74
△FVC (L)			− 0.09 (− 0.20 to 0.01)	0.04 (− 0.11 to 0.14)	0.06	0.03 (− 0.13 to 0.10)	− 0.06 (− 0.10 to 0.05)	0.88
△FEV_1_ (L)			− 0.05 (− 0.20 to 0.00)	0.02 (− 0.04 to 0.11)	0.01	0.07 (− 0.01 to 0.21)	0.03 (− 0.05 to 0.05)	0.32
△FEF_25–75%_ (L/s)			− 0.35 (− 0.52 to 0.11)	0.11 (− 0.34 to 0.50)	0.02	1.03 (0.27 to 1.40)	− 0.09 (− 0.34 to 0.32)	0.06
△*D*_LCO_ (% predicted value)			− 4.50 (− 10.25 to 0.00)	− 0.50 (− 9.00 to 2.00)	0.33	− 6.00 (− 10.00 to − 0.50)	8.00 (1.50 to 10.75)	0.10
△R_5_–R_20_ (kPa L(− 1)sec)			0.02 (− 0.01 to 0.04)	0.00 (− 0.03 to 0.03)	0.10	0.00 (− 0.01 to 0.03)	0.01 (− 0.01 to 0.03)	0.95
△X_5_ (kPa L(− 1)sec)			− 0.01 (− 0.03 to 0.02)	0.00 (− 0.04 to 0.03)	0.81	− 0.03 (− 0.06 to 0.00)	− 0.03 (− 0.04 to 0.01)	0.62
△AX (kPa L(− 1))			0.08 (− 0.07 to 0.29)	0.03 (− 0.21 to 0.18)	0.63	0.07 (− 0.05 to 0.22)	0.11 (0.05 to 0.20)	0.63
△Fres (Hz)			− 0.13 (− 0.92 to 1.75)	− 0.17 (− 1.36 to 1.50)	0.85	0.93 (0.12 to 1.65)	1.29 (0.48 to 2.11)	0.84
△CAT score			1.00 (− 1.25 to 5.25)	− 3.00 (− 7.00 to − 1.00)	0.01	1.00 (− 2.00 to 2.50)	4.50 (1.75 to 6.25)	0.22
**△SGRQ**			4.61 (− 4.26 to 12.31)	− 1.28 (− 11.70 to 6.37)	0.15	− 1.14 (− 6.14 to 2.10)	− 1.55 (− 6.63 to 3.86)	0.65
△Symptom domain			− 0.21 (− 4.18 to 2.85)	− 5.33 (− 16.46 to 11.03)	0.77	− 14.54 (− 23.89 to − 5.16)	3.05 (− 7.91 to 15.56)	0.11
△Activity domain			13.28 (− 2.23 to 32.38)	0.32 (− 12.36 to 11.83)	0.07	0.00 (− 14.54 to 14.44)	− 2.92 (− 9.30 to 1.46)	0.57
△Impact domain			2.06 (− 4.47 to 6.83)	− 1.67 (− 13.64 to 5.46)	0.32	0.00 (− 2.81 to 4.85)	4.40 (− 3.33 to 8.71)	0.78

Small airway dysfunction (SAD) is defined as AX > 0.44 (kPa/L) measured by impulse oscillometry. The data are described as median (interquartile range, IQR) for non-normally distributed variables. *p* values were generated from the Mann–Whitney U test for two-group comparisons.

*BD Rx* bronchodilator treatment, *FVC* forced vital capacity, *FEV1* forced expiratory volume in the 1st second, *FEF*_*25–75%*_ forced expiratory flow after expiration of 25% to 75% of forced vital capacity, *DLCO* diffusing capacity for carbon monoxide, *R5* resistance at 5 Hz, *R20* resistance at 20 Hz, *X5* reactance at 5 Hz, *Fres* resonant frequency, *AX* area of reactance, *CAT* COPD assessment test, *SGRQ* St. George Respiratory Questionnaire, *△* difference between visit 1 and visit 2.

**Table 6:** Bronchodilator efficacy in patients with SAD defined according to different cutoffs of IOS parameters.

		AX > 0.44	R_5_–R_20_ > 0.07	X5 < − 0.12	Fres > 14.14
Δ FVC (L)		0.04 (0.06)	0.06 (0.08)	0.04 (0.05)	0.05 (0.14)
Δ FEV_1_ (L)		0.02 (0.01)	0.02 (0.02)	0.03 (0.02)	0.02 (0.12)
Δ FEF_25–75%_ (L/s)		0.11 (0.02)	0.19 (0.01)	0.06 (0.19)	0.05 (0.25)
Δ *D*_LCO_ (% predicted value)		− 0.50 (0.33)	− 0.50 (0.23)	0.00 (0.20)	0.00 (0.43)
Δ R_5_-R_20_ (kPa L(− 1)sec)		0.00 (0.10)	− 0.02 (0.04)	0.00 (0.07)	0.00 (0.09)
Δ X_5_ (kPa L(− 1)sec)		0.00 (0.81)	0.00 (0.97)	0.00 (0.96)	− 0.02 (0.88)
Δ AX (kPa L(− 1))		0.03 (0.63)	− 0.01 (0.74)	0.02 (0.39)	0.04 (0.67)
Δ Fres (Hz)		− 0.17 (0.85)	− 0.17 (0.80)	− 0.17 (0.31)	− 0.10 (0.91)
Δ CAT score		− 3.00 (0.01)	− 2.00 (0.01)	− 3.00 (< 0.01)	− 2.00 (0.01)
**Δ SGRQ**		− 1.28 (0.15)	− 1.28 (0.34)	− 1.28 (0.18)	0.68 (0.11)
Δ Symptom domain		− 5.33 (0.77)	− 5.33 (0.67)	− 5.97 (0.64)	− 3.39 (0.94)
Δ Activity domain		0.32 (0.07)	0.00 (0.36)	0.32 (0.05)	0.00 (0.11)
Δ Impact domain		− 1.67 (0.32)	− 1.67 (0.40)	− 0.19 (0.64)	− 0.69 (0.27)

The data are described as difference (Δ) followed by the *p*-value.

*△* difference between visit 1 and visit 2, *FVC* forced vital capacity, *FEV*_*1*_ forced expiratory volume in the 1st second, *FEF*_*25–75%*_ forced expiratory flow after expiration of 25% to 75% of forced vital capacity, *DLCO* diffusing capacity for carbon monoxide, *R5* resistance at 5 Hz, *R20* resistance at 20 Hz, *X5* reactance at 5 Hz, *Fres* resonant frequency, *AX* area of reactance, *CAT* COPD assessment test, *SGRQ* St. George Respiratory Questionnaire.

## Discussion

SAD exists in various bronchiolar and interstitial lung diseases, including asthma and COPD^28^. IOS has high sensitivity to detect peripheral airway obstruction in an effort-independent way^29^. We demonstrated that the IOS parameters may be useful to guide bronchodilator therapy in patients with IPF who have coexisting SAD. IPF patients treated with bronchodilators according to the IOS parameter AX showed significant improvement in FEV_1_, FEF_25–75%_, and symptom score after bronchodilator treatment compared to those without bronchodilator treatment. Patients with SAD defined according to R_5_–R_20_ and X_5_ had similar benefits from bronchodilator treatment. Bronchodilator efficacy was not observed in patients without SAD. There was no significant improvement in lung function or symptom score after bronchodilator treatment in patients with emphysema. IOS parameters appear to be a potential guide for bronchodilator treatment in IPF patients with SAD.

IPF may be comorbid with obstructive lung diseases. Assayag et al.^11^ reported that in a large cohort, nearly one in ten patients with IPF had physiological evidence of reversible airflow limitation. Smoking appears to be the major risk factor for the development of COPD and IPF^30^. In this study, 35% of patients had combined pulmonary fibrosis and emphysema, most of whom were current or ex-smokers. The Spanish guidelines for the treatment of IPF suggest that for patients with obstructive or mixed functional limitations, inhaled bronchodilators may be prescribed, as for COPD^31^. The French guidelines propose that inhaled bronchodilators should be used if airflow obstruction is present in patients with IPF and emphysema^10^. FEV_1_/FVC < 0.7 indicates airflow obstruction and is therefore a criterion for the use of inhaled bronchodilators in IPF. This is no longer practical, as most patients with IPF have FEV_1_/FVC > 0.8, as shown both here and in some large clinical trials^32,33^. In this study, the FEV_1_, FVC, *D*_LCO_, and IOS parameters and symptom scores were not different between groups with and without emphysema. FEV_1_/FVC and FEF_25–75%_ were lower in the IPF with emphysema group. Even in IPF patients with emphysema, the median FEV_1_/FVC was still 0.81. In addition, the bronchodilator efficacy was not observed in patients with emphysema. Therefore, we can conclude that emphysema is not a deciding factor in prescribing bronchodilator treatment in IPF patients.

IOS is a noninvasive and effort-independent procedure using several frequencies of sound waves to measure the resistance and reactance of the airways. R_5_–R_20_, indicating small airway resistance, is currently the key IOS parameter applied for diagnosing SAD in patients with asthma, COPD, or environmental exposure^34,35^. The correlations between IOS parameters (R_5_–R_20_, Fres and AX) and spirometric measurements (FEV_1_, FVC and FEF_25–75%_) were significant in subjects with respiratory symptoms and preserved pulmonary function^27^. In IPF, as structural alterations occur in the distal bronchioles and alveolar regions, lung volume, diffusing capacity and conducting airway resistance are lowered^6^. Increases in FEV_1_/FVC and FEF_25–75%_/FVC as well as the increase in airway dimensions at all lung depths have been observed in IPF^36^. However, investigations assessing small airway function in IPF are scarce. In this study, we found that small airway resistance and reactance were higher in patients with IPF than in normal healthy subjects; these were determined according to the IOS parameters R_5_–R_20_, X_5_, AX and Fres^27^. These findings are consistent with those reported by Sugiyama et al.^37^. The increase in FEF_25–75%_ is consistent with other findings^30^. Only AX was significantly correlated with FEV_1_ (% predicted value), FEF_25–75%_ (% predicted value) and SGRQ activity domain score in patients with IPF. R_5_–R_20_ and X5 did not have similar correlations. AX has also been used to detect early rejection in patients with lung transplant^38^. In a study of hypersensitive pneumonitis, AX was elevated in all patients and lung volume improved after treatment^39^. AX may therefore be a useful marker along with R_5_–R_20_ to indicate SAD.

SGRQ and CAT were originally developed to measure the health status of COPD patients. The SGRQ total score is an independent prognostic factor in IPF^17^. CAT is also a valid health status measurement in IPF, and it shows significant correlations with dyspnea severity, oxygenation impairment, and anxiety. The CAT score significantly correlates with the total SGRQ score^20^. Regarding asthma, a recent study reported that the association between FEV_1_% and asthma control questionnaire (ACQ) scores was weak^40^. In COPD, the CAT score has a weak negative correlation with FEV_1_%, suggesting individual variation in these measures. The correlations between symptom scores and lung function parameters were poor in this study. However, the differences in FEV_1_ and CAT score in patients with and without SAD were 70 mL (+ 0.02L vs. − 0.05L) and 4 points (+ 1.00 vs. − 3.00), respectively, both with statistical significance (*p* = 0.01). Although the overall SGRQ score did not show a significant difference, there was a trend (*p* = 0.07) showing improvement on the activity domain after bronchodilator treatment in IPF patients with SAD. To date, the minimal clinically important difference (MCID) has not been evaluated in patients with IPF^41^. There is some evidence of a MCID between different outcomes in pharmacological trials of COPD, including 100 mL for FEV_1_, 2 points for CAT score, and 4 units for SGRQ^42,43^. Our results demonstrated improvement in both lung function and symptom burden, which had statistical significance when the IOS-defined SAD patients received bronchodilator treatment. The change in the CAT score, which reflects patients’ symptoms, reached the MCID according to the existing evidence. On the other hand, the drop in oxygen saturation during the 6MWT was significantly associated with FVC, FEV_1_, *D*_LCO_, SGRQ score, and SGRQ activity domain score. The levels of desaturation during exercise comprise extended parenchymal fibrosis, alterations of ventilation, and the hemodynamics and abnormality of gas exchange. In addition, exertional desaturation is associated with physical activity and mortality in IPF^44^. Exertional desaturation during walking could be more sensitive and objective than symptom scores.

The limitations of this study include its retrospective design and the small number of patients in each subgroup, which may have reduced the statistical power to detect differences in lung function and IOS parameters at baseline and during follow-up. Other limitations were the unidentified factors, such as COPD, asthma, and other small airway diseases, that may have increased airway resistance and reactance; the lack of cutoff values of the IOS parameters AX, R_5_–R_20_, Fres, and X_5_ for IPF patients without coexisting SAD; and finally the possible influence of lung volume improvement on other IPF outcomes (i.e. exacerbations), which needs a longer follow-up period to answer. The strength of this study is that it provides a useful tool to detect SAD in IPF and guide bronchodilator therapy.

## Conclusion

In conclusion, the FEV_1_/FVC ratio cannot reflect the true airflow obstruction in IPF as it is masked by reduced lung volume. Emphysema in IPF is not a deciding factor for whether patients should receive bronchodilator treatment. IOS parameters, which indicate small airway function, may be useful for guiding bronchodilator therapy.

## Supplementary information

Supplementary Information.

## Data Availability

The datasets generated during and/or analyzed during the current study are not publicly available because they contain personal information with privacy concerns, but are available from the corresponding author on reasonable request.

## References

[1] Travis WD (2013). An official American Thoracic Society/European Respiratory Society statement: Update of the international multidisciplinary classification of the idiopathic interstitial pneumonias. Am. J. Respir. Crit. Care Med..

[2] Bagnato G, Harari S (2015). Cellular interactions in the pathogenesis of interstitial lung diseases. Eur. Respir. Rev..

[3] Raghu, G. *et al.* Diagnosis of idiopathic pulmonary fibrosis. An official ATS/ERS/JRS/ALAT clinical practice guideline. *Am. J. Respir. Crit. Care Med.* **198,** e44–e68 (2018). 10.1164/rccm.201807-1255ST 30168753

[4] Raghu G (2011). An official ATS/ERS/JRS/ALAT statement: Idiopathic pulmonary fibrosis: Evidence-based guidelines for diagnosis and management. Am. J. Respir. Crit. Care Med..

[5] Marshall, D. C., Salciccioli, J. D., Shea, B. S. & Akuthota, P. Trends in mortality from idiopathic pulmonary fibrosis in the European Union: An observational study of the WHO mortality database from 2001–2013. *Eur. Respir. J.* **51** (2018). 10.1183/13993003.01603-2017 29348182

[6] Plantier, L. *et al.* Physiology of the lung in idiopathic pulmonary fibrosis. *Eur. Respir. Rev.* **27** (2018). 10.1183/16000617.0062-2017 PMC9489199 29367408

[7] Raghu G, Amatto VC, Behr J, Stowasser S (2015). Comorbidities in idiopathic pulmonary fibrosis patients: A systematic literature review. Eur. Respir. J..

[8] Pastre J (2015). Different KCO and VA combinations exist for the same DLCO value in patients with diffuse parenchymal lung diseases. BMC Pulm. Med..

[9] *2020 Global Strategy for Prevention, Diagnosis and Management of COPD.* https://goldcopd.Org/gold-reports/. Accessed 10 Mar 2020.

[10] Cottin V (2014). Diagnosis and management of idiopathic pulmonary fibrosis: French practical guidelines. Eur. Respir. Rev..

[11] Assayag D (2015). The effect of bronchodilators on forced vital capacity measurement in patients with idiopathic pulmonary fibrosis. Respir. Med..

[12] Bickel S, Popler J, Lesnick B, Eid N (2014). Impulse oscillometry: Interpretation and practical applications. Chest.

[13] Anderson WJ, Zajda E, Lipworth BJ (2012). Are we overlooking persistent small airways dysfunction in community-managed asthma?. Ann. Allergy Asthma Immunol..

[14] Crisafulli E (2017). Prevalence of small-airway dysfunction among COPD patients with different GOLD stages and its role in the impact of disease. Respiration.

[15] Jones, P. W., Quirk, F. H., Baveystock, C. M. & Littlejohns, P. A self-complete measure of health status for chronic airflow limitation: The St. George’s Respiratory Questionnaire. *Am. Rev. Respir. Dis.* **145,** 1321–1327 (1992). 10.1164/ajrccm/145.6.1321 1595997

[16] Jones PW (2009). Development and first validation of the COPD assessment test. Eur. Respir. J..

[17] Furukawa, T. *et al.* The St. George’s Respiratory Questionnaire as a prognostic factor in IPF. *Respir. Res. 18* (2017). 10.1186/s12931-017-0503-3 PMC5240376 28095852

[18] Kreuter M (2017). Health related quality of life in patients with idiopathic pulmonary fibrosis in clinical practice: insights-IPF registry. Respir. Res..

[19] Kreuter M (2020). Health-related quality of life and symptoms in patients with IPF treated with nintedanib: Analyses of patient-reported outcomes from the INPULSIS trials. Respir. Res..

[20] Matsuda T (2017). COPD assessment test for measurement of health status in patients with idiopathic pulmonary fibrosis: A cross-sectional study. Respirology.

[21] Grufstedt HK, Shaker SB, Konradsen H (2018). Validation of the COPD assessment test (CAT) in patients with idiopathic pulmonary fibrosis. Eur. Clin. Respir. J..

[22] Graham, B. L. *et al.* Standardization of spirometry 2019 update. An official American Thoracic Society and European Respiratory Society technical statement. *Am. J. Respir. Crit. Care Med.* **200,** e70–e88 (2019). 10.1164/rccm.201908-1590ST PMC6794117 31613151

[23] Graham, B. L. *et al.* 2017 ERS/ATS standards for single-breath carbon monoxide uptake in the lung. *Eur. Respir. J.* **49** (2017). 10.1183/13993003.00016-2016 28049168

[24] ATS Committee on Proficiency Standards for Clinical Pulmonary Function Laboratories. ATS statement: Guidelines for the six-minute walk test. *Am. J. Respir. Crit. Care Med.* **166,** 111–117 (2002). 10.1164/ajrccm.166.1.at1102 12091180

[25] Pellegrino R (2005). Interpretative strategies for lung function tests. Eur. Respir. J..

[26] Oostveen E (2003). The forced oscillation technique in clinical practice: Methodology, recommendations and future developments. Eur. Respir. J..

[27] Chiu H-Y (2020). Small airway dysfunction by impulse oscillometry in symptomatic patients with preserved pulmonary function. J. Allergy Clin. Immunol. Pract..

[28] Ryu JH, Myers JL, Swensen SJ (2003). Bronchiolar disorders. Am. J. Respir. Crit. Care Med..

[29] Brashier B, Salvi S (2015). Measuring lung function using sound waves: Role of the forced oscillation technique and impulse oscillometry system. Breathe (Sheff).

[30] Morse D, Rosas IO (2014). Tobacco smoke-induced lung fibrosis and emphysema. Annu. Rev. Physiol..

[31] Xaubet A (2017). Guidelines for the medical treatment of idiopathic pulmonary fibrosis. Arch. Bronconeumol..

[32] Richeldi L (2014). Efficacy and safety of nintedanib in idiopathic pulmonary fibrosis. N. Engl. J. Med..

[33] King TE (2014). A phase 3 trial of pirfenidone in patients with idiopathic pulmonary fibrosis. N. Engl. J. Med..

[34] Lipworth B, Manoharan A, Anderson W (2014). Unlocking the quiet zone: The small airway asthma phenotype. Lancet Respir. Med..

[35] Haruna A (2010). Relationship between peripheral airway function and patient-reported outcomes in COPD: A cross-sectional study. BMC Pulm. Med..

[36] Brand P (1999). Aerosol-derived airway morphometry and aerosol bolus dispersion in patients with lung fibrosis and lung emphysema. Chest.

[37] Sugiyama A (2013). Characteristics of inspiratory and expiratory reactance in interstitial lung disease. Respir. Med..

[38] Ochman M (2018). Usefulness of the impulse oscillometry system in graft function monitoring in lung transplant recipients. Transplant. Proc..

[39] Guerrero Zúñiga, S. *et al.* Small airway dysfunction in chronic hypersensitivity pneumonitis. *Respirology* **22,** 1637–1642 (2017). 10.1111/resp.13124 28748646

[40] Sullivan PW, Ghushchyan VH, Marvel J, Barrett YC, Fuhlbrigge AL (2019). Association between pulmonary function and asthma symptoms. J. Allergy Clin. Immunol. Pract..

[41] Canu S, Alfieri V, Renzoni E (2017). Patient-reported outcome measures in idiopathic pulmonary fibrosis: Where do we stand?. Respirology.

[42] Jones, P.W. *et al.* Minimal clinically important differences in pharmacological trials. *Am. J. Respir. Crit. Care Med.* **189**, 250–255 (2014). 10.1164/rccm.201310-1863PP 24383418

[43] Kon, S.S. *et al.* Minimum clinically important difference for the COPD assessment test: A prospective analysis. *Lancet Respir. Med.* **2**, 195–203 (2014). 10.1016/S2213-2600(14)70001-3 24621681

[44] Vainshelboim, B., Kramer, M. R., Izhakian, S., Lima, R. M. & Oliveira, J. Physical activity and exertional desaturation are associated with mortality in idiopathic pulmonary fibrosis. *J. Clin. Med. Res.* **5** (2016). 10.3390/jcm5080073 PMC4999793 27548238

