# Supplementary material for: Functional parameters of small airways can guide bronchodilator use in idiopathic pulmonary fibrosis
Source: Sci Rep. 2020 Oct 29;10:18633. doi: 10.1038/s41598-020-75597-2 (PMC7596229; doi:10.1038/s41598-020-75597-2)
Supplement: Supplementary file 1 — Supplementary Information. [file 41598_2020_75597_MOESM1_ESM.docx]

**Supplementary Information**

**Functional parameters of small airways can guide bronchodilator use in idiopathic pulmonary fibrosis**

Po-Wei Hu,^1^ Hsin-Kuo Ko,^1, 2^ Kang-Cheng Su,^1, 2, 3^ Jia-Yih Feng,^1,2^ Wei-Juin Su,^1,2^ Yi-Han Hsiao,^1, 2, 3^* & Diahn-Warng Perng^1, 2^*

^1^ Department of Chest Medicine, Taipei Veterans General Hospital, Taipei, Taiwan, R.O.C.

^2^ Faculty of Medicine, School of Medicine, National Yang-Ming University, National Yang-Ming University, Taipei, Taiwan, R.O.C.

^3^ Department of Physiology, School of Medicine, National Yang-Ming University, Taipei, Taiwan, R.O.C.

* Yi-Han Hsiao and Diahn-Warng Perng corresponded equally to this study.

**Supplementary Methods**

**Pulmonary function tests**

Because forced expiration could affect the IOS parameters including resistance and reactance, spirometry was conducted immediately after the IOS examination. All patients were advised to avoid inhaled bronchodilators 12 hours before spirometry. Two flow-sensing spirometers each connected to a computer (Jaeger MS-IOS^®^, Germany and Vmax 22 SensorMedics, Yorba Linda, CA, USA) for data analysis were used. From the forced expiratory flow-volume curve, we measured FEF_25%-75%_, FVC and FEV_1_. The percentage of predicted indices mention above were also obtained. The equipment was calibrated twice a day and the quality of spirometry was confirmed by two independent pulmonology specialists with assistance from a third specialist in case of disagreement.

**Impulse oscillometry**

Prior to the IOS examinations, no premedication was allowed. Nasal clips and manual compression of the cheeks were used to reduce the confounding factors of cheek vibration and escape of air via the nostrils. The tested subject breathed quietly through the mouthpiece with the loudspeaker activated, for 30–45 s without glottis contraction or interposition of the tongue. During that period the loudspeaker emitted about 120–150 pulses that varied between 5 and 35 Hz in frequency and overlapped the normal breathing sounds (0.2–2 Hz). We evaluated the following IOS parameters: difference of resistance at 5Hz and 20 Hz (R_5_–R_20_), reactance at 5Hz (X_5_), resonant frequency (Fres), and area under reactance curve between 5 Hz and resonant frequency (AX). Morphology of R or X graphs was also taken into consideration. The equipment was calibrated twice a day.

**Supplementary Tables**

**Table S1.** The correlation between symptom scores and lung function

|  | | SGRQ | Activity domain | CAT |
| --- | --- | --- | --- | --- |
| FVC | (% predicted value) | -0.131 (0.330) | -0.206 (0.123) | -0.181 (0.179) |
| FEV_1_ | (% predicted value) | -0.017 (0.899) | -0.051 (0.706) | -0.173 (0.198) |
| FEF_25%-75%_ | (% predicted value) | 0.125 (0.365) | 0.203 (0.138) | -0.018 (0.900) |
| *D*_LCO_ | (% predicted value) | 0.280 (0.035) | -0.394 (0.002) | -0.129 (0.337) |

The data are described using Pearson correlation coefficient (r) followed by *p*-value. SGRQ: St. George Respiratory Questionnaire; CAT: COPD assessment test, 6MWT: six-minute walk test; FEV1: forced expiratory volume in the 1st second; FVC: forced vital capacity; FEF25%-75%: forced expiratory flow after expiration of 25% to 75% of forced vital capacity; DLCO: diffusing capacity for carbon monoxide

**Table S2.** The correlations between lung function or symptom scores and IOS parameters or changes in SaO_2_

|  | | | R_5_-R_20_ | X_5_ | AX | Fres | △SaO_2_ |
| --- | --- | --- | --- | --- | --- | --- | --- |
| FVC | | (% predicted value) | -0.112 (0.381) | 0.213 (0.094) | -0.181 (0.156) | -0.196 (0.124) | -0.408 (<0.001) |
| FEV_1_ | | (% predicted value) | -0.182 (0.154) | 0.261 (0.039) | -0.263 (0.037) | -0.178 (0.164) | -0.316 (0.012) |
| FEF_25%-75%_ | | (% predicted value) | -0.199 (0.124) | 0.193 (0.135) | -0.288 (0.024) | -0.201 (0.121) | 0.019 (0.886) |
| *D*_LCO_ | | (% predicted value) | 0.003 (0.982) | 0.058 (0.650) | -0.076 (0.553) | -0.130 (0.309) | -0.354 (0.005) |
| SGRQ | | | 0.266 (0.046) | -0.121 (0.369) | 0.257 (0.054) | 0.243 (0.068) | 0.323 (0.014) |
|  | Activity domain | | 0.284 (0.032) | -0.124 (0.356) | 0.268 (0.044) | 0.311 (0.019) | 0.403 (0.002) |
| CAT score | | | 0.220 (0.101) | -0.173 (0.198) | 0.252 (0.058) | 0.269 (0.043) | 0.251 (0.059) |

The data are described using Pearson correlation coefficient (r) followed by *p* value. FVC: forced vital capacity; FEV1: forced expiratory volume in the 1st second; FEF25%-75%: forced expiratory flow after expiration of 25% to 75% of forced vital capacity; DLCO: diffusing capacity for carbon monoxide; SGRQ: St. George Respiratory Questionnaire; CAT: COPD assessment test; R5: resistance at 5 Hz; R20: resistance at 20 Hz; X5: reactance at 5 Hz; Fres: resonant frequency; AX: area of reactance; △SaO2: difference between highest and lowest oxygen saturation (%) during six-minute walk test (6MWT) at baseline (1^st^ visit).

**Table S3.** The bronchodilator efficacy in patients with SAD defined by R_5_-R_20_ > 0.07(kPa L(-1)sec)

|  | | SAD (+)  (N = 40) | | | SAD (-)  (N = 23) | | |
| --- | --- | --- | --- | --- | --- | --- | --- |
|  | | BD Rx (-)  (N = 16) | BD Rx (+)  (N = 24) | *p* value | BD Rx (-)  (N = 9) | BD Rx (+)  (N = 14) | *p* value |
| △FVC (L) | | -0.07  (-0.22 to 0.04) | 0.06  (-0.08 to 0.13) | 0.08 | -0.09  (-0.15 to 0.09) | -0.05  (-0.11 to 0.13) | 0.73 |
| △FEV_1_ (L) | | -0.04  (-0.24 to 0.01) | 0.02  (-0.05 to 0.12) | 0.02 | 0.03  (-0.05 to 0.12) | 0.03  (-0.03 to 0.06) | 0.78 |
| △FEF_25%-75%_ (L/sec) | | -0.36  (-0.55 to -0.05) | 0.19  (-0.33 to 0.47) | 0.01 | 0.59  (0.23 to 1.17) | -0.05  (-0.49 to 0.44) | 0.10 |
| △*D*_LCO_ (% predicted value) | | -4.50  (-14.50 to 0.00) | -0.50  (-9.00 to 1.25) | 0.23 | -5.00  (-9.00 to 2.00) | 1.50  (-6.50 to 9.00) | 0.30 |
| △R_5_-R_20_ (kPa L(-1)sec) | | 0.03  (-0.01 to 0.04) | -0.02  (-0.04 to 0.01) | 0.04 | 0.00  (-0.01 to 0.04) | 0.01  (0.00 to 0.03) | 0.31 |
| △X_5_ (kPa L(-1)sec) | | -0.01  (-0.02 to 0.04) | 0.00  (-0.04 to 0.04) | 0.97 | -0.03  (-0.05 to 0.00) | -0.03  (-0.04 to 0.00) | 0.49 |
| △AX (kPa L(-1)) | | 0.02  (-0.14 to 0.27) | -0.01  (-0.30 to 0.17) | 0.74 | 0.07  (-0.02 to 0.31) | 0.11  (0.04 to 0.21) | 1.00 |
| △Fres (Hz) | | -0.13  (-1.04 to 1.23) | -0.17  (-1.74 to 1.15) | 0.80 | 0.93  (-0.55 to 1.94) | 0.54  (-0.68 to 2.08) | 0.64 |
| △CAT score | | 1.00  (-0.75 to 4.75) | -2.00  (-7.00 to -1.00) | 0.01 | 2.00  (-3.00 to 3.00) | -2.00  (-5.25 to 3.25) | 0.32 |
| △SGRQ | | 2.47  (-6.84 to 10.28) | -1.28  (-11.43 to 6.1) | 0.34 | 1.41  (-1.70 to 12.30) | -1.20  (-10.90 to 6.98) | 0.46 |
|  | △Symptom domain | -0.21  (-5.02 to 3.26) | -5.33  (-16.46 to 4.95) | 0.67 | -7.63  (-19.85 to -2.69) | 2.37  (-9.26 to 14.46) | 0.13 |
|  | △Activity domain | 6.07  (-15.4 to 23.75) | 0.00  (-11.21 to 11.62) | 0.36 | 11.16  (0.00 to 30.88) | 2.90  (-19.48 to 7.60) | 0.10 |
|  | △Impact domain | 2.06  (-2.53 to 8.47) | -1.67  (-10.86 to 5.46) | 0.40 | -0.05  (-5.57 to 5.81) | -0.05  (-14.20 to 7.40) | 0.81 |

The data are described as difference (**Δ**) followed by *p*-value, △: difference between visit 1 and visit 2; FVC: forced vital capacity; FEV_1_:forced expiratory volume in the 1^st^ second; FEF_25%-75%_: forced expiratory flow after expiration of 25% to 75% of forced vital capacity; DLCO: diffusing capacity for carbon monoxide; R5: resistance at 5 Hz; R20: resistance at 20 Hz; X5: reactance in 5 Hz; Fres: resonant frequency; AX: area of reactance; CAT: COPD assessment test; SGRQ: St. George Respiratory Questionnaire.

**Table S4.** The bronchodilator efficacy in patients with SAD defined by X_5_ < 0.12(kPa L(-1)sec)

|  | | SAD (+)  (N = 46) | | | SAD (-)  (N = 17) | | |
| --- | --- | --- | --- | --- | --- | --- | --- |
|  | | BD Rx (-)  (N = 18) | BD Rx (+)  (N = 28) | *p* value | BD Rx (-)  (N = 7) | BD Rx (+)  (N = 10) | *p* value |
| △FVC (L) | | -0.09  (-0.20 to 0.01) | 0.04  (-0.08 to 0.15) | 0.05 | 0.03  (-0.13 to 0.15) | -0.01  (-0.14 to 0.09) | 0.77 |
| △FEV_1_ (L) | | -0.05  (-0.20 to 0.02) | 0.03  (-0.03 to 0.11) | 0.02 | 0.03  (-0.04 to 0.10) | 0.03  (-0.07 to 0.05) | 0.56 |
| △FEF_25%-75%_ (L/sec) | | -0.23  (-0.52 to 0.14) | 0.06  (-0.54 to 0.49) | 0.19 | 0.59  (-0.21 to 1.10) | 0.09  (-0.18 to 0.47) | 0.38 |
| △*D*_LCO_ (% predicted value) | | -4.50  (-10.25 to 0.00) | 0.00  (-9.00 to 2.00) | 0.20 | -6.00  (-10.00 to -0.50) | 2.50  (-6.75 to 10.75) | 0.33 |
| △R_5_-R_20_ (kPa L(-1)sec) | | 0.02  (-0.01 to 0.04) | 0.00  (-0.03 to 0.02) | 0.07 | 0.00  (-0.01 to 0.03) | 0.01  (-0.01 to 0.03) | 0.77 |
| △X_5_ (kPa L(-1)sec) | | -0.01  (-0.03 to 0.02) | 0.00  (-0.04 to 0.03) | 0.96 | -0.03  (-0.06 to 0.00) | -0.03  (-0.04 to 0.01) | 0.88 |
| △AX (kPa L(-1)) | | 0.08  (-0.07 to 0.29) | 0.02  (-0.27 to 0.15) | 0.39 | 0.07  (-0.05 to 0.22) | 0.15  (0.04 to 0.28) | 0.47 |
| △Fres (Hz) | | 0.03  (-0.85 to 2.44) | -0.17  (-1.56 to 1.15) | 0.31 | 0.79  (-0.58 to 1.16) | 1.29  (-0.54 to 3.76) | 0.42 |
| △CAT score | | 1.50  (-0.50 to 5.25) | -3.00  (-7.00 to -1.00`) | < 0.01 | 0.00  (-2.50 to 2.00) | -1.00  (-2.25 to 4.25) | 0.91 |
| △SGRQ | | 4.61  (-4.26 to 12.31) | -1.28  (-15.13 to 6.37) | 0.18 | 1.41  (-6.14 to 2.54) | -2.55  (-10.83 to 6.50) | 0.96 |
|  | △Symptom domain | -1.68  (-5.99 to 0.78) | -5.97  (-16.53 to 11.03) | 0.64 | -14.54  (-23.89 to 2.68) | 5.44  (-4.82 to 14.00) | 0.12 |
|  | △Activity domain | 13.28  (-0.19 to 32.38) | 0.32  (-12.36 to 11.62) | 0.05 | -11.16  (-18.11 to 14.44) | -0.23  (-9.23 to 7.55) | 0.96 |
|  | △Impact domain | 0.36  (-4.47 to 4.64) | -0.19  (-10.86 to 7.15) | 0.64 | 0.00  (-2.79 to 11.63) | -1.67  (-14.40 to 4.58) | 0.32 |

The data are described as difference (**Δ**) followed by *p*-value, △: difference between visit 1 and visit 2; FVC: forced vital capacity; FEV_1_:forced expiratory volume in the 1^st^ second; FEF_25%-75%_: forced expiratory flow after expiration of 25% to 75% of forced vital capacity; DLCO: diffusing capacity for carbon monoxide; R5: resistance at 5 Hz; R20: resistance at 20 Hz; X5: reactance in 5 Hz; Fres: resonant frequency; AX: area of reactance; CAT: COPD assessment test; SGRQ: St. George Respiratory Questionnaire.

**Table S5.** The bronchodilator efficacy in patients with SAD defined by Fres>14.14Hz

|  | | SAD (+)  (N = 52) | | | SAD (-)  (N = 11) | | |
| --- | --- | --- | --- | --- | --- | --- | --- |
|  | | BD Rx (-)  (N = 17) | BD Rx (+)  (N = 35) | *p* value | BD Rx (-)  (N = 8) | BD Rx (+)  (N = 3) | *p* value |
| △FVC (L) | | -0.04  (-0.15 to 0.09) | 0.05  (-0.09 to 0.15) | 0.14 | -0.10  (-0.18 to 0.05) | -0.11  (-0.17 to -0.08) | 0.68 |
| △FEV_1_ (L) | | -0.02  (-0.17 to 0.03) | 0.02  (-0.06 to 0.11) | 0.12 | -0.05  (-0.20 to 0.08) | 0.03  (0.02 to 0.05) | 0.63 |
| △FEF_25%-75%_ (L/sec) | | -0.10  (-0.42 to 0.14) | 0.05  (-0.35 to 0.47) | 0.25 | 0.13  (-0.68 to 1.07) | 0.45  (0.02 to 0.71) | 0.92 |
| △*D*_LCO_ (% predicted value) | | -1.00  (-8.00 to 1.00) | 0.00  (-9.00 to 2.50) | 0.43 | -7.50  (-11.75 to -4.50) | 6.00  (0.50 to 8.00) | 0.08 |
| △R_5_-R_20_ (kPa L(-1)sec) | | 0.01  (-0.01 to 0.04) | 0.00  (-0.03 to 0.02) | 0.09 | 0.01  (-0.01 to 0.04) | 0.04  (0.03 to 0.06) | 0.18 |
| △X_5_ (kPa L(-1)sec) | | -0.02  (-0.03 to 0.02) | -0.02  (-0.04 to 0.03) | 0.88 | 0.00  (-0.03 to 0.01) | 0.02  (0.01 to 0.03) | 0.38 |
| △AX (kPa L(-1)) | | 0.07  (-0.08 to 0.30) | 0.04  (-0.13 to 0.21) | 0.67 | 0.03  (-0.07 to 0.24) | 0.08  (0.06 to 0.10) | 0.78 |
| △Fres (Hz) | | -0.28  (-0.93 to 1.18) | -0.10  (-1.22 to 1.63) | 0.91 | 1.16  (0.44 to 2.30) | 2.18  (1.26 to 4.24) | 0.5 |
| △CAT score | | 2.00  (-0.50 to 4.50) | -2.00  (-6.50 to 0.50) | 0.01 | 0.50  (-3.25 to 2.00) | -4.00  (-4.50 to -3.50) | 0.24 |
| △SGRQ | | 2.66  (-1.60 to 11.51) | 0.68  (-11.57 to 7.03) | 0.11 | -3.55  (-10.92 to 5.17) | -7.32  (-8.98 to -5.67) | 0.71 |
|  | △Symptom domain | -0.42  (-4.6 to 1.31) | -3.39  (-15.76 to 13.20) | 0.94 | -17.2  (-22.83 to -1.04) | -9.91  (-10.56 to -9.26) | 0.71 |
|  | △Activity domain | 6.21  (-3.72 to 30.39) | 0.00  (-15.40 to 11.72) | 0.11 | 8.68  (-18.02 to 21.01) | -0.24  (-3.27 to 2.80) | 0.71 |
|  | △Impact domain | 3.62  (-1.17 to 9.79) | -0.69  (-12.25 to 7.65) | 0.27 | -2.79  (-8.15 to 1.45) | -9.10  (-13.69 to -4.50) | 0.69 |

The data are described as difference (**Δ**) followed by *p*-value, △: difference between visit 1 and visit 2; FVC: forced vital capacity; FEV_1_:forced expiratory volume in the 1^st^ second; FEF_25%-75%_: forced expiratory flow after expiration of 25% to 75% of forced vital capacity; DLCO: diffusing capacity for carbon monoxide; R5: resistance at 5 Hz; R20: resistance at 20 Hz; X5: reactance in 5 Hz; Fres: resonant frequency; AX: area of reactance; CAT: COPD assessment test; SGRQ: St. George Respiratory Questionnaire.
